# Why low-intensity endurance training for athletes?

**DOI:** 10.1007/s00421-025-05843-w

**Published:** 2025-06-27

**Authors:** Pekka Matomäki

**Affiliations:** 1 Paavo Nurmi Centre & Unit for Health and Physical Activity, University of Turku, Turku, Finland; 2 Faculty of Sport and Health Sciences, University of Jyväskylä, Jyväskylä, Finland

**Keywords:** Low-intensity training, Elite athlete, Endurance training, Endurance performance

## Abstract

Endurance athletes prioritize most of their training in low-intensity zone. This forms a paradox, as immediate logic would argue against it: Acutely low-intensity exercise does not challenge the homeostasis or cardiopulmonary system of high-level athletes sufficiently to produce performance gains comparable to those from moderate- or high-intensity exercise. In this perspective study, seven possible explanations for the purpose of excessive-volume low-intensity training in endurance athletes are proposed. The hypotheses are not all mutually exclusive. They range from a psychological need for easy days and the incremental benefits of low-intensity training without accumulating stress, to the possibility that such training may ultimately be replaceable.

## Introduction

There is a compelling paradox: Why do endurance athletes spend so much time on low-intensity training when logic suggests it should no longer lead to further improvements?

Endurance training intensities can be categorized into three zones delineated by two lactate/ventilation thresholds: low- (LI), moderate- (MI), and high-intensity (HI) zones. When compared to other zones, low-intensity exercises do not significantly challenge the cardiopulmonary system, disturb the homeostatic balance, or cause metabolic perturbation (Poole and Jones 2012). At least not for individuals with prior training history. Since such challenges, disruptions, and perturbations typically trigger the most prominent endurance training stimuli (Hoppeler 2016; Odden et al. 2024) low-intensity training is typically regarded inferior compared to high-intensity training in relation to performance gain (Midgley et al. 2006).

**Fig. 1:**
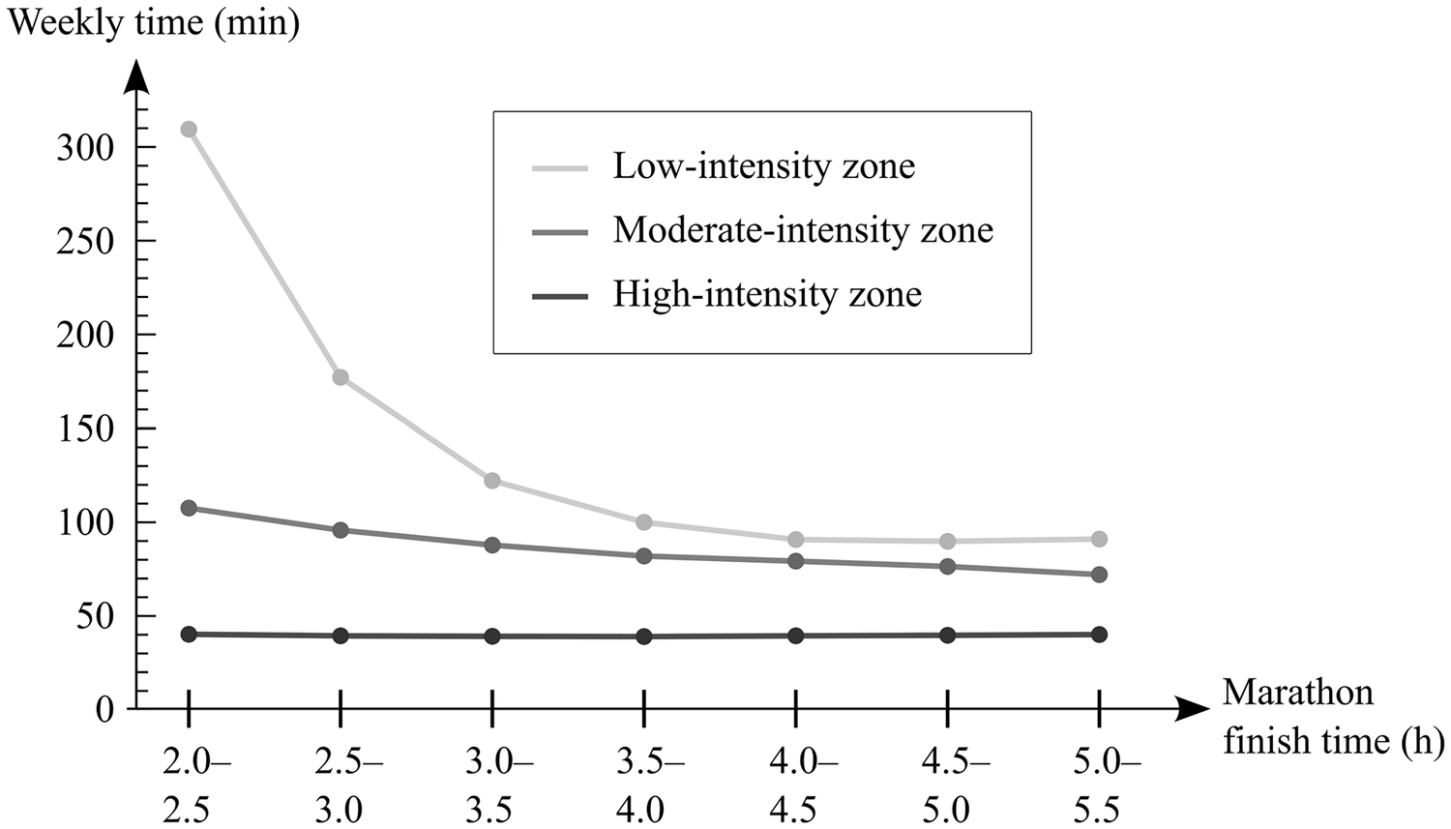
Mean absolute external training time in different zones over the 16 weeks leading up to the marathon, categorized by marathon finish time. Moderate- and high-intensity training time are not drastically different between the groups. A sample of 100,020 male runner participants. Data extracted from Muniz-Pumares et al. (2024)

In the context of this article, an ‘athlete’ is defined as at least Tier 3 in the 0- to 5 -classification (McKay et al. 2022), meaning they compete at least at the national level, are within 20% of the world record and engage in structured and periodized training while developing towards (within 20% of) maximal norms. Despite research indicating the relevance of high-intensity training in athletes (Midgley et al. 2006; Wen et al. 2019; Odden et al. 2024), goal-oriented athletes seem to almost universally prioritize the volume of low-, and not high-, intensity training in their programs. For example, low-intensity training volume appears to distinguish high-level runners from slower ones (Fig. 1). Consequently, elite athletes commonly devote the majority of their time to the low-intensity zone, with isolated cases that may even exceed 90% (Sperlich et al. 2023).

If we assume that modern sports training has evolved over decades toward optimal programming, the question arises: What is the independent purpose of LI training and why athletes choose to do so much of it? In this perspective, seven hypotheses are presented to address this apparent illogicality. The hypotheses are summarized in Table 1. At the end of the study, the role of low-intensity training for untrained individuals is briefly reflected. Athletes rarely participate in invasive comparative studies, leading to small sample sizes and predominantly observational research. Therefore, much of the theory behind the hypotheses is based on research with recreationally active individuals.

**Table 1:** Summary of hypotheses of why endurance athletes should engage in excess amount of low-intensity training

1. It provides maintenance or slight improvements to performance without cumulating stress
2. It is an alternative method for molecular adaptation signals
3. It enables structural remodeling after years of consistency
4. It affects something that has not yet been measured
5. It is needed psychologically
6. It strengthens high-intensity training adaptations
7. LI training is replaceable

These hypotheses are not all mutually exclusive

Terminological note: In this study, *exercise* is defined as a single planned endurance training session. Performing multiple exercises systematically over an extended period is defined as *training*.

### Hypothesis 1

LI training provides maintenance or slight improvements to performance without cumulating stress.

Complete cardiac autonomic recovery from HI exercises might take over two days (Stanley et al. 2013), limiting athletes to 2–3 weekly well-rested HI sessions. This leaves room for weekly 4–5 days that could be filled with LI training. Cardiac autonomic recovery from a typical LI exercise occurs in < 24 h (Stanley et al. 2013), sometimes even within hours (Seiler et al. 2007). Thus, LI exercises seem to contribute only to a limited degree to cardiac autonomic strain accumulation. While this represents only one dimension of recovery, it nevertheless suggests that LI exercises may be repeated more frequently than HI sessions. However, LI training has been observed to benefit performance to some extent in recreationally trained individuals (Nuuttila et al. 2022). This could potentially indicate that it has the capacity to enhance performance for athletes, as also suggested by some of the leading exercise physiologists (Sitko et al. 2025).

In summary, LI training might maintain, or modestly improve, performance while allowing recovery from HI exercises.

### Hypothesis 2

LI training is an alternative method for molecular adaptation signals.

At the muscle level, some endurance adaptations, such as mitochondrial biogenesis, are thought to be triggered by peroxisome proliferator-activated receptor gamma coactivator 1 (PGC-1α). This key regulator is reported to be activated through various means, including free radicals, increased Ca^2+^ flux, changes in hormonal homeostasis, and increased metabolic stress caused by energy deficiency, glucose deficiency, or elevated fatty acid concentrations (Hoppeler 2016).

It might be that both LI and HI exercises activate PGC-1$\alpha$, but are initiated through different pathways. It has been suggested that HI training activates primarily metabolic pathways, while LI training affects fatty acid and Ca^2+^ -routes (Hoppeler 2016), although not everyone agrees (Gurd et al. 2023). In other words, LI training might provide an alternative adaptation pathway and, in doing so, introduce variability into the training adaptation process. Variability, in turn, is one of the fundamental principles underpinning functional endurance periodization (Kiely 2012).

### Hypothesis 3

LI training enables structural remodeling after years of consistency.

Training interventions typically last < 4 months. However, cross-sectional studies show that over 5 years of consistent endurance training leads to greater physiological adaptations compared to < 2 years (Buzza 2018). These differences include improved oxygen utilization at the muscle level and higher maximal oxygen uptake (VO_2max_) values (Buzza 2018).

Determining whether the additional long-term adaptations result specifically from high-volume LI training is challenging, as they may also result from a synergistic combination of LI, MI, and HI training or other factors like nutrition and recovery or genetics. However, indirect evidence suggests LI training might play a role. An analysis of over 150,000 marathoners found that while weekly absolute MI and HI training times remained relatively stable regardless of marathon performance, high-level runners had by far the greatest LI training time (Muniz-Pumares et al. 2024). Thus, it is possible that consistent high-volume LI training result in structural adaptations that separate champions from amateurs, although the effects of well-timed and sufficiently intense MI and HI exercises, genetics, and sufficient nutrition and rest cannot be ruled out.

This ‘structural hypothesis’ is not a new proposition. Some of the leading experts have also suggested LI training to predispose athletes to peripheral and even subtle central adaptations (Sitko et al. 2025). LI training may be involved, among other factors, in the following structural changes.

### Structure of the heart

LI training might be involved to gradually remodel the structure and function of the left ventricle. To support this view, the diameter of the left ventricular has increased during the careers of professional cyclists (Abergel et al. 2004). Additionally, it has been hypothesized that the pericardium, the sac surrounding the heart, may limit cardiac output (Esch et al. 2007). According to this hypothesis, high-volume LI training gradually helps to stretch the pericardium, allowing the heart to enlarge over time.

### Capillaries

Angiogenesis is driven mainly by shear stress and muscle stretch (Hoppeler 2016), both present not only during HI but also during LI exercise. This suggests that high-volume LI training could be part of promoting capillary formation over years.

### Muscle fiber ratio

Animal studies show that 24-h daily electric shocks can shift muscle composition to primarily slow-twitch fibers (Schiaffino and Reggiani 2011), which are better suited for endurance activities than fast-twitch fibers. It can be theorized that high-volume endurance training, whether LI, MI, or HI, may similarly facilitate gradual remodeling. In humans, such adaptation through natural training methods would likely take years. Supporting this, an increased proportion of slow-twitch fibers has been observed in cross-country skiers after several years of training (Rusko 1992).

Compared to fast-twitch fibers, slow-twitch ones are more economical (Krustrup et al. 2008), so a gradual increase in their proportion may play a role in the gradual improvement in movement economy observed in elite athletes over the course of their careers (Jones 2006), although also changes in anthropometric, biomechanical, and other physiological factors affects to the movement economy (Jones 2006).

### Mitochondrial mass

It has been suggested that HI training could improve the efficiency of individual mitochondria to produce energy, while high-volume training could be linked to an increase in total mitochondrial mass (Bishop et al. 2014). Thus, HI and LI training may target different aspects of mitochondrial biogenesis.

#### Hypothesis 4

LI training affects something that has not yet been measured.

Endurance performance comprises multiple components, including fat oxidation capacity, threshold intensities, oxygen kinetics, movement economy, durability, and VO_2max_ (Jones 2023). Among these, VO_2max_ has been the most extensively studied and has been thought to be linked with HI training in athletes (Midgley et al. 2006; Wen et al. 2019; Odden et al. 2024). However, the effect of training on other components remains less understood, leaving room for the potential role of excessive LI training. Indeed, a high volume of LI training has recently been associated with good durability (Spragg et al. 2023).

Recovery is another surprisingly overlooked variable. While athletes recover from submaximal exercises faster than recreationally active individuals (Seiler et al. 2007), it is unclear which type of training optimally affects recovery ability. It might be that high volume of LI training could be involved in improving the ability to recover more swiftly after an exercise session and also to maintain homeostasis in exercise (Issurin and Dreshman 2012).

In summary, years of dedicated LI training may be involved in improving components that are not yet fully understood or clearly defined.

#### Hypothesis 5

LI training is needed psychologically.

Acutely, LI exercise improves mood in both untrained and trained individuals, while HI exercise decreases it (Berger and Motl 2000). Moreover, in athletes, short-term LI training decreases mental fatigue (Faude et al. 2009), while HI training block increases distress (Halson et al. 2002). In summary, emphasized MI or HI training might simply be psychologically too demanding.

#### Hypothesis 6

LI training strengthens HI training adaptations.

Walking and high step count alone may not be sufficient to increase VO_2max_ at least in individuals with some training background (Swain and Franklin 2002). However, combining a high total volume of low-intensity physical activity with HI training appears to be more effective than HI training alone in untrained (Burton et al. 2021) and moderately trained individuals (Hautala et al. 2012). The hypothesis in this context is that physical activity, in the form of LI training within athletic programming, helps the body not to ‘resist’ training adaptation process (Burton et al. 2021).

#### Hypothesis 7

LI training is replaceable.

One option is that, with appropriate programming and training dose control, the volume of LI training could be dramatically reduced and replaced by higher-intensity training. A concern with this approach is the increased risk of overtraining syndrome and a higher injury rate.

While overtraining syndrome may be witnessed in the field, very little objective scientific data exists on it (Weakley et al. 2022), and even that limited data may be confounded by insufficient energy intake (Stellingwerff et al. 2021). Therefore, it might be that with sufficient training dose control and recovery days and weeks, overtraining syndrome could be avoided. However, without an appropriate recovery plan, intensified training can lead to a fatigued state and a reduction in cardiac function (Hatle et al. 2014).

Additionally, high intensity during exercises increases peak forces and thus increases the risk of sudden-onset overload injuries compared to low intensity (Boullosa et al. 2020). On the flip side, low-intensity exercises are longer and involve a higher number of repetitions, which also increases the risk of overuse injuries (Boullosa et al. 2020). Therefore, one cannot unequivocally state that intensity or volume is a more detrimental stressor for injury.

Given these considerations, emphasized MI or HI training might be considered feasible training programming. In fact, there are examples where a year-long HI-focused training has not been at least counterproductive for athletes (Gaskill et al. 1999). Moreover, such training has been shown to be both superior (Evertsen et al. 1999) and inferior (Stöggl and Sperlich 2014) to traditional training programming for athletes.

Indeed, the comparison between intensified training and traditional polarized/pyramidal training involving a high volume of LI training reveals no clear difference in performance improvements, whereas VO_2max_ could be better improved by traditional training (Silva Oliveira et al. 2024).

Athletes may be hesitant to abandon LI training, as they have learned to rely on it and it has proved to be effective. However, it might be that there is not a strict necessity to include a high volume of LI training after all. Carefully planned intensified training could be an equally feasible training method as traditional methods incorporating a high volume of LI training.

Should LI training turn out to be replaceable by more intensified training, it would suggest that top-level athletic performance depends not on the large volume of LI training, but rather on a large volume of *endurance* training, regardless of intensity.

## Reflections

High training volume itself, typically accumulated through low-intensity training, is associated with better endurance performance (Muniz-Pumares et al. 2024). Thus, although the exact rationale behind the high volume of low-intensity training is not fully understood, it appears to be effective.

Much of the theory behind the presented hypotheses is based on research with recreationally active individuals. This may distort perspectives, as elite athletes and recreationally active individuals represent different populations. For example, while training at an intensity of 50% VO_2max_ can further improve VO_2max_ in recreationally active individuals (Swain and Franklin 2002), it seems that international-level athletes may need over 90% VO_2max_ intensities for further gains (Odden et al. 2024).

The training dose of a training program increases with volume. Therefore, a justified question is whether LI training is still ‘easy’ training when performed in large volumes, even exceeding 15 weekly hours in athletes? Overall, evidence suggests that a few hours long LI sessions are easily tolerated by well-trained athletes, as measured by blood lactate concentration, rating of perceived exertion, and cardiac autonomic recovery time (Seiler et al. 2007; Almquist et al. 2020), at least compared to individuals with more modest training background (Seiler et al. 2007; Matomäki et al. 2023). This is at least partly due to their superior durability, which prevents deterioration in physiological profiling characteristics during prolonged efforts (Jones 2023).

## Prescribing intensity zones

In this perspective, the ‘low-intensity’ -zone was determined by physiological thresholds. This is sensible, as the origin of fatigue in the physiological LI zone shifts from being centrally driven to also including a peripheral component as intensity extends into the MI zone (Black et al. 2017). This is evident in the greater fatigability in the MI zone compared to the LI zone (Brownstein et al. 2022).

However, in real life, the training intensity may be dictated using intrinsic threshold values, such as heart rate, or external ones, such as velocity. Internal variables may have kinetics, meaning their response to intensity changes is delayed compared to external variables. Additionally, internal measures are seldom constant throughout an exercise session, but change in response to increasing physiological strain, as illustrated for example by cardiac drift in heart rate. Moreover, the training intensities can be anchored to race-pace instead of thresholds complicating the intensity distribution calculations.

Compared to heart rate-controlled intensity distribution, velocity control tends shift the distribution toward the HI zone, possibly at the expense of the LI zone (Bellinger et al. 2020; Matzka et al. 2022). In the race-pace approach, the emphasis on the LI zone may not differ from threshold velocity-based approaches (Kenneally et al. 2021; Matzka et al. 2022). However, dividing intensities into zones based on race-pace is somewhat arbitrary, making the approach prone to different interpretations. Nevertheless, no matter how intensity is prescribed, a consistent pattern tends to emerge across all measurement methods at the group level: The LI zone, defined through the physiological threshold, appears to be the predominant training zone, although the exact emphasis varies (Ranieri et al. 2025). This underscores that the physiological LI zone remains a key component of training, regardless of how the pace of low-intensity sessions is prescribed.

## Low-intensity training for untrained

For untrained individuals, no paradox of LI training arises. LI training most likely challenges the cardiopulmonary system of untrained individuals by maximizing stroke volume (Spina et al. 1992). As a result, LI training is sufficient on its own to improve performance markers such as VO_2max_ and threshold intensity (Nuuttila et al. 2025). From a health perspective, maintaining for example metabolic risk markers within a healthy range is often sufficient for untrained people, without the need for the same level of optimization required for athletic performance.

In addition, for most untrained individuals, physical activity and exercising may not be seen as an optimization problem but rather as a form of social interaction, an enjoyable hobby, or a way to maintain fitness and health (Allender et al. 2006). Therefore, there is typically no paradox regarding why LI training should not be prioritized for untrained individuals.

## Conclusion

While athletes are eager to fill their weeks with low-intensity training, the exact reason for its use remains elusive. In this perspective study, seven hypotheses are presented. Most likely, a single clear reason for low-intensity training, or its redundancy, cannot be found; rather, the truth is likely a combination of several explanations.
